# THOC5/FMIP, an mRNA export TREX complex protein, is essential for hematopoietic primitive cell survival *in vivo*

**DOI:** 10.1186/1741-7007-8-1

**Published:** 2010-01-05

**Authors:** Annalisa Mancini, Susanne C Niemann-Seyde, Rüdiger Pankow, Omar El Bounkari, Sabine Klebba-Färber, Alexandra Koch, Ewa Jaworska, Elaine Spooncer, Achim D Gruber, Anthony D Whetton, Teruko Tamura

**Affiliations:** 1 Institut fuer Biochemie, OE4310, Medizinische Hochschule Hannover, Carl-Neuberg-Str. 1, D-30623 Hannover, Germany; 2 Abteilung Molekulare Genetik, Forschungsinstitut fuer Molekulare Pharmakologie, Krahmerstr. 6, D-12207 Berlin, Germany; 3 Stem Cell and Leukemia Proteomics Laboratory, Manchester Academic Health Sciences Centre, University of Manchester, Christie Hospital, Manchester M20 4BX, UK; 4 Institute of Veterinary Pathology, Freie Universitaet Berlin, Robert-von-Ostertag- Str. 15, D-14163 Berlin, Germany; 5 Developmental and Regenerative Biology, Mount Sinai School of Medicine, One Gustave L. Levy Place, New York, NY 10029, USA

## Abstract

**Background:**

The transcription/export complex is evolutionarily conserved from yeast to man and is required for coupled transcription elongation and nuclear export of mRNAs. FMIP(Fms interacting protein) is a member of the THO (suppressors of the transcriptional defects of hpr1delta by overexpression) complex which is a subcomplex of the transcription/export complex. THO complex (THOC) components are not essential for bulk poly (A)+ RNA export in higher eukaryotes, but for the nuclear export of subset of mRNAs, however, their exact role is still unclear.

**Results:**

To study the role of THOC5/Fms interacting protein *in vivo*, we generated THOC5/Fms interacting protein knockout mice. Since these mice are embryonic lethal, we then generated interferon inducible conditional THOC5/Fms interacting protein knockout mice. After three poly injections all of the mice died within 14 days. No pathological alterations, however, were observed in liver, kidney or heart. Thus we considered the hematopoietic system and found that seven days after poly injection, the number of blood cells in peripheral blood decreased drastically. Investigation of bone marrow cells showed that these became apoptotic within seven days after poly injection. Committed myeloid progenitor cells and cells with long term reconstituting potential were lost from bone marrow within four days after poly injection. Furthermore, infusion of normal bone marrow cells rescued mice from death induced by loss of THOC5/Fms interacting protein.

**Conclusion:**

THOC5/Fms interacting protein is an essential element in the maintenance of hematopoiesis. Furthermore, mechanistically depletion of THOC5/Fms interacting protein causes the down-regulation of its direct interacting partner, THOC1 which may contribute to altered THO complex function and cell death.

## Background

During expression of protein-coding genes, pre-mRNAs are transcribed in the nucleus and undergo several RNA-processing steps. The mature mRNA is then exported from the nucleus to the cytoplasm for translation. Nuclear export of mRNA composes one part of a larger network of molecular events that begin with transcription of the mRNA in the nucleus and end with its translation and degradation in the cytoplasm. The TREX (transcription/export) complex is conserved in evolution from yeast to man and is required for coupled transcription elongation and nuclear export of mRNAs [1-4]. The TREX complex in mammals and *Drosophila *is composed of the THO (suppressors of the transcriptional defects of hpr1delta by overexpression) subcomplex (THO-complex 1 (THOC1) 1, THOC2, THOC5, THOC6 and THOC7), THOC3, UAP56 and Aly/THOC4 [5,6]. However, the THO complex components are not essential for bulk poly (A) + RNA export in higher eukaryotes [7-10]. Furthermore, the nuclear export of only a subset of mRNAs is affected by depletion of a member of the THO complex [5,10]. These data suggest that various nuclear mRNA export pathways, which may be indicated by different adaptor RNA binding proteins, exist in higher eukaryotes. Recent data show that Aly and THOC5 function in the tip associating protein (TAP)-p15 mediated nuclear export of Hsp70 mRNA [11]. It was demonstrated that the depletion of THOC5 does not affect bulk poly (A)+ RNA export, but does affect Hsp70 mRNA export in Hela cells. Interestingly, the deletion of THOC1, a major conserved component of THO complex, causes apoptosis in transformed cells, but not in normal fibroblasts [12]. Furthermore, the embryonic development of the conventional THOC1 knockout mice is arrested around the time of implantation [13], suggesting that the THO complex may play an essential role in early development.

Fms interacting protein (FMIP) was originally identified as a substrate for the Macrophage Colony Stimulating Factor (M-CSF) receptor tyrosine kinase, Fms [14]. FMIP has been demonstrated to be a member of THO complex, THOC5 [5,6]. We have previously shown that depletion of THOC5/FMIP by siRNA or ectopic expression causes abnormal hematopoiesis and abnormal adipocyte differentiation in myeloid progenitor or mesenchymal progenitor cell lines, indicating that the THO-complex is essential for the differentiation process in mammals [14-17].

In this study we show that THOC5/FMIP is essential at an early stage of murine development. Furthermore, using interferon inducible THOC5/FMIP knockout mice we demonstrated that this gene is essential for survival. In these mice, bone marrow and spleen cells became apoptotic, hematopoietic progenitor cell numbers collapsed and the animals became anemic. Although the THOC5/FMIP gene was deleted in liver, kidney, and heart, pathological alterations to these organs were not observed. Furthermore, 9 out of 14 THOC5/FMIP depleted mice survived over two months by normal bone marrow cell transplantation with no apparent symptoms.

## Results

### THOC5/FMIP is essential at an early stage of mouse development

**Figure 1:**
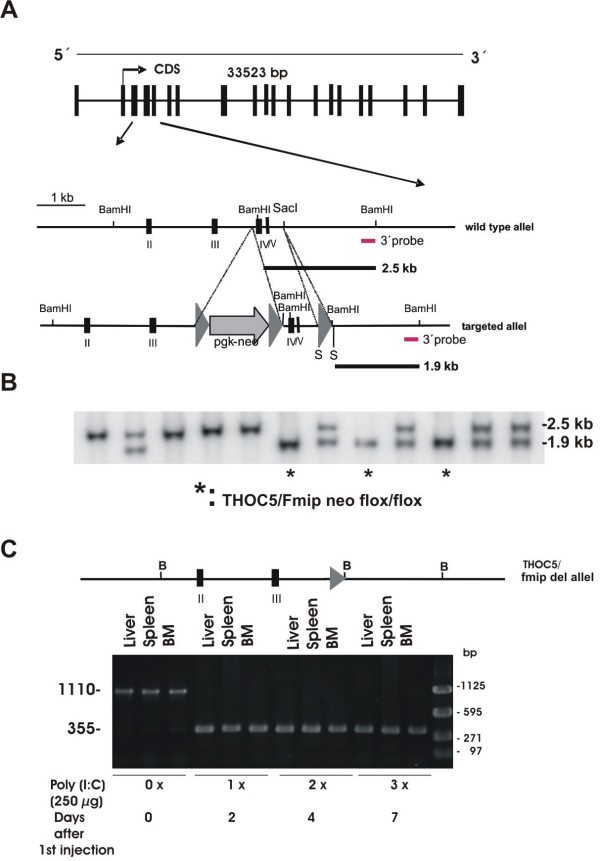
**Generation of THOC5/FMIP (flox/flox) and Mx-cre THOC5/FMIP (flox/flox) mice**. **(A) **The genomic structure of the 33,523 kb THOC5/FMIP locus is depicted. THOC5/FMIP deficient mice were generated using a genomic DNA fragment containing exons II, III, IV, and V isolated from 129/ola genetic background. The targeting vector was constructed from a 3.9 kb PCR fragment and an adjacent 2.2 kb fragment harboring exons IV/V. Fragments were introduced into the pPNTloxPneo vector via NotI, XhoI restriction sites and KpnI, respectively. An additional loxP site was inserted into the SacI site downstream of exon V: **(B) **After transfection, ES cell clones carrying the inserted floxed neo cassette and floxed Fmip exons IV/V were identified by the presence of an 1.9 kb BamHI restriction fragment in Southern Blot analysis with external 3'probe ((*) THOC5/fmip neo flox/flox). **(C) **Mx-cre THOC5/FMIP (flox/flox) were sacrificed before (zero day) and after two (one × poly (I:C) injection), four (two × poly (I:C) injection) and seven (three × poly (I:C) injection) days after the first poly (I:C) injection and genomic DNAs were extracted from liver, spleen, and bone marrow. Genomic DNAs were supplied for the deletion of exons IV/V determined by PCR using 5'-TGCTGGCATTGAACTGTG-3' and 5'-CAGCACTGGAGCGGGAGATGT-3'. PCR product: wild type allel: 1110 bp; THOC5/FMIP del allele: 355 bp.

To examine the role of THOC5/FMIP *in vivo*, we first generated a floxed THOC5/FMIP allele (THOC5/FMIP flox) by recombination in embryonic stem (ES) cells [18]. Given that the THOC5/FMIP gene spans 20 exons in a 33,523 kb region on chromosome 11, we adopted a targeting strategy where, by flanking exons IV and V with loxP sites, we could inactivate THOC5/FMIP in a conditional manner (Figure 1A). The deletion of exons IV/V of THOC5/FMIP causes a frame shift of product and the truncated protein is expected to be only 83 amino acids long and lacking the THOC1 binding domain [19]. ES cells were used to establish a THOC5/FMIP flox strain (Figure 1A and 1B). Homozygous THOC5/FMIP (flox/flox) mice were fertile and did not display any phenotypic or histological abnormalities, showing that the two loxP sites in the THOC5/FMIP flox locus did not affect its function. First, we bred THOC5/FMIP *floxed *mice to EIIa-Cre mice in which Cre is ubiquitously expressed [20]. After generation of heterozygote mice (F1), we bred F1 mice and generated F2 mice. We did not detect any THOC5/FMIP-/- newborn mice from F2 mice. In addition, the genotype of 59 embryos collected between E5.5 and E8.5 was determined by the exon IV/V specific PCR analysis of genomic DNA. Of 59 embryos which we examined, none was THOC5/FMIP-/- indicating that loss of THOC5/FMIP causes embryonic lethality before E5.5. Among 59 embryos, 18 were THOC5/FMIP +/+ and 41 were +/- which was the expected Mendelian ratio (*P *< 0.0001 by Chi squared test). THOC5/FMIP +/- mice were indistinguishable from wild type.

To examine THOC5/FMIP depletion in adult mice, THOC5/FMIP (flox/flox) and cre-*deleter *mice expressing cre-recombinase (cre) under the control of the interferon-inducible Mx promoter (Mx-cre) [21] were crossed to obtain animals that carry the THOC5/FMIP(flox/flox) allele and Mx-cre gene (Mx-cre THOC5/FMIP (flox/flox)).

### THOC5/FMIP expression is reduced in specific organs

The deletion mutation of THOC5/FMIP was induced by poly (I:C) injection. We first injected six-week-old Mx-cre THOC5/FMIP (flox/flox) (n = 6) with 250 μg of poly (I:C) three times, at two to three day intervals. Mx-cre THOC5/FMIP (flox/flox) mice (two mice each) were sacrificed before and two (one × poly (I:C) injection), four (two × poly (I:C) injection) and seven (three × poly (I:C) injection) days after the first poly (I:C) injection and genomic DNAs were extracted from liver, spleen and bone marrow. Genomic DNAs were used for the determination of exon IV/V deletion by PCR using 5'-TGCTGGCATTGAACTGTG-3' and 5'-CAGCACTGGAGCGGGAGATGT-3' (Figure 1C). To our surprise, PCR product lacking exons VI/V (355 bp) was detected in all organs after one × poly (I:C) injection in all cases (Figure 1C).

Then, we injected six-week-old Mx-cre THOC5/FMIP (flox/flox) (n = 8) and control THOC5/FMIP(flox/flox) (n = 6) mice with 250 μg of poly (I:C) three times, at two to three days intervals. Mx-cre THOC5/FMIP (flox/flox) (n = 4 each) and control THOC5/FMIP(flox/flox) (n = 3 each) mice were sacrificed at four and seven days after the first poly (I:C) injection and proteins were extracted from liver, spleen, kidney, lung, intestine, testicles and bone marrow. It has been reported that the treatment of the interferon mediated Cre-inducible mice with poly (I:C) causes deletion of the target gene only in particular organs, such as liver and spleen, and to a lesser extent in heart and kidney [21].

**Figure 2:**
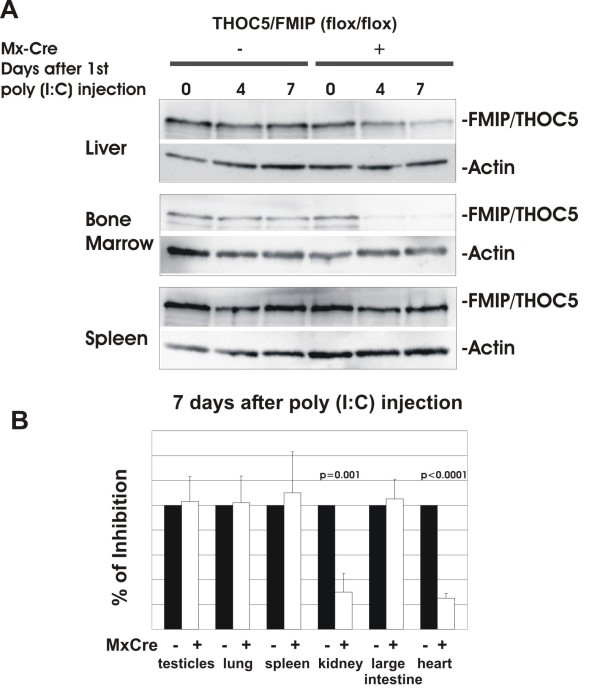
**THOC5/FMIP expression is reduced in specific organs**. **(A, B) **250 μg of poly (I:C) were injected into six-week-old Mx-cre THOC5/FMIP (flox/flox) (n = 8) or control THOC5/FMIP (flox/flox) (n = 6) mice three times at two-to-three-day intervals. THOC5/FMIP protein levels in liver, bone marrow, spleen (A), testicles, lung, kidney, large intestine, and heart (B) were examined by THOC5/FMIP specific immunoblotting. Proteins were extracted from each organ. Total protein amount was standardized by actin specific immunoblotting.

We examined, therefore, the THOC5/FMIP protein level in liver, spleen, kidney, lung, intestine, testicles and bone marrow using THOC5/FMIP specific antibody and western blot [16]. After poly (I:C) injection, the level of THOC5/FMIP is reduced by approximately 80% in liver (*P *< 0.0001), kidney (*P *= 0.001), heart (*P *< 0.0001) and bone marrow (*P *< 0.0001) within seven days in all Mx-cre THOC5/FMIP (flox/flox) mice. In contrast, in spleen, intestine, testes and lung, the level is not reduced (Figure 2A and 2B).

### Deletion of THOC5/FMIP gene causes death within two weeks

**Figure 3:**
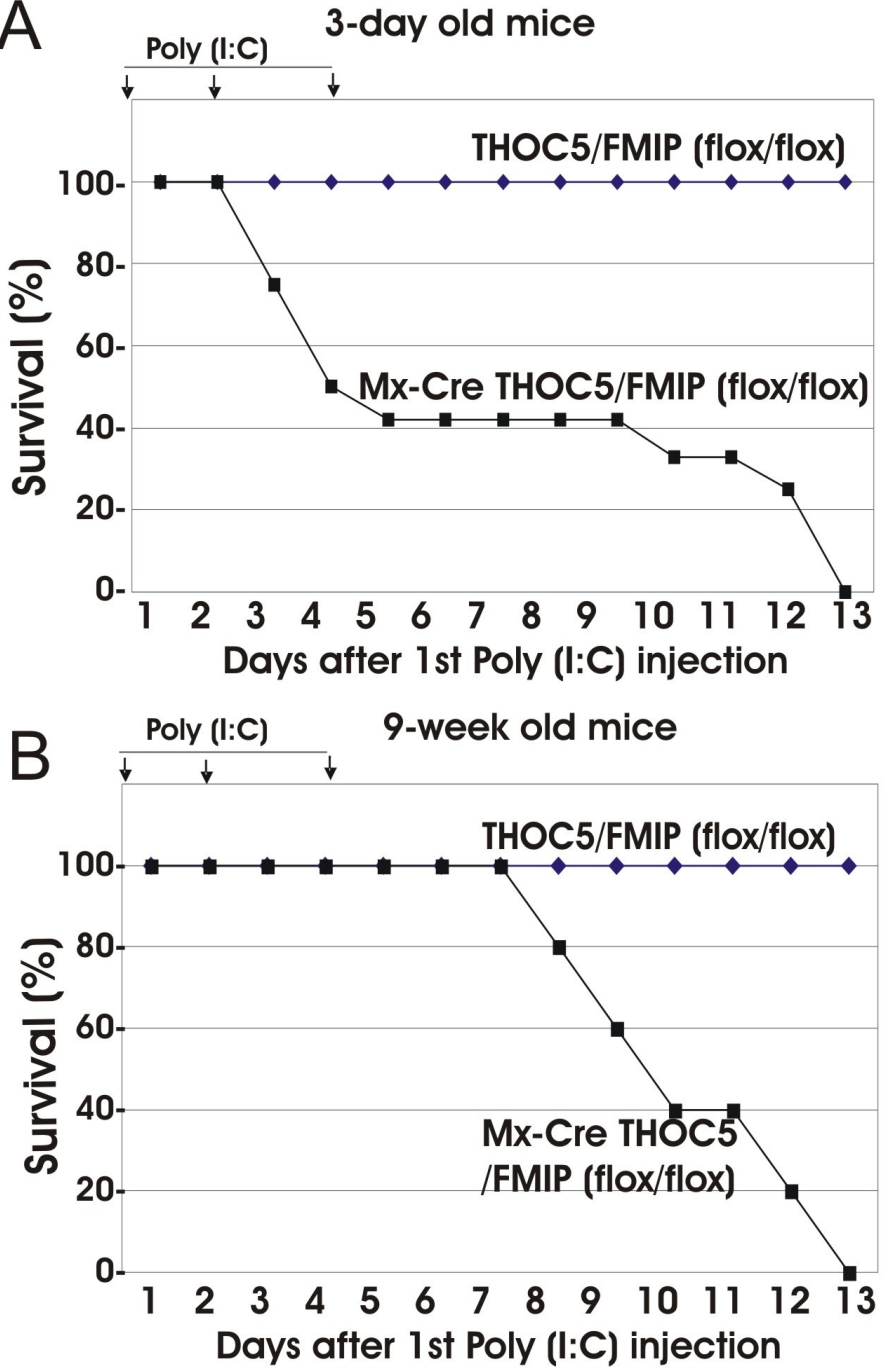
**Deletion of THOC5/FMIP is lethal in adult mice**. Three day- (n = 25) **(A) **and nine-week-old (n = 7) **(B) **mice were injected with 50, and 500 μg of poly (I:C), respectively. Injection was performed i.p. three times at two to three day intervals. (arrows: poly (I:C) injection).

Since conventional knockout mice died at the embryonic stage, we determined whether THOC5/FMIP is necessary for postnatal mouse survival. We therefore injected three-day-old mice (n = 25) with 50 μg of poly (I:C) three times at two-to-three-day intervals. Mx-cre THOC5/FMIP(flox/flox) mice began dying three days after injection and then all died within 14 days after the first injection, whereas all control mice (THOC5/FMIP(flox/flox)) survived (Figure 3A). We then injected 500 μg of poly (I:C) into nine-week-old mice (n = 7) three times over a two-to-three-day interval. These mice began dying eight days after the first poly (I:C) injection and all of them were dead within 14 days (Figure 3B), indicating that THOC5/FMIP gene expression is essential for the survival of adult mice.

**Figure 4:**
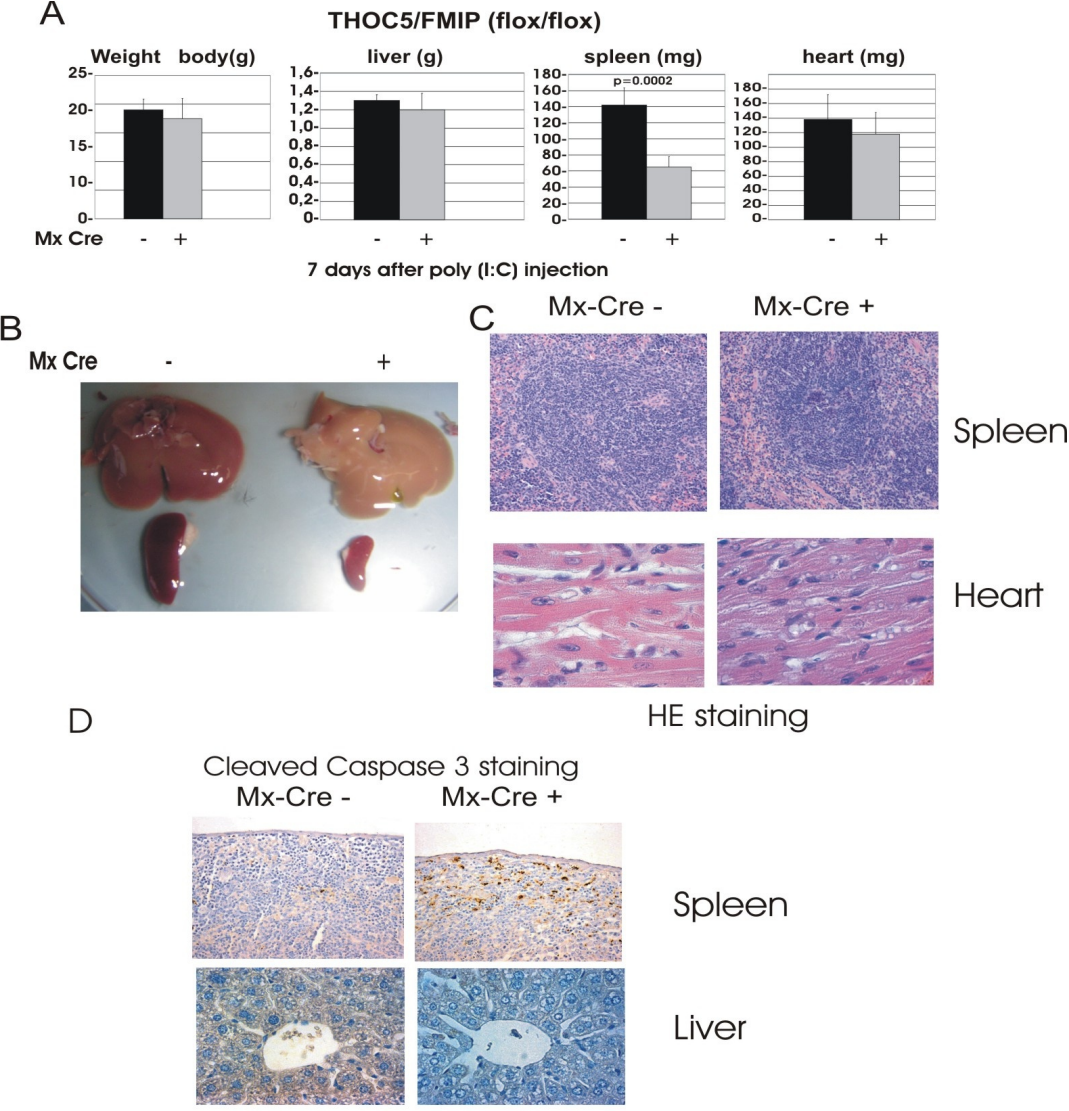
**THOC5/FMIP deletion causes reduction of spleen size and induction of cleaved caspase 3**. Two hundred and fifty micrograms of poly (I:C) were injected into six-week-old Mx-cre THOC5/FMIP (flox/flox) (n = 9) or control THOC5/FMIP (flox/flox) (n = 6) mice three times at two-to-three-day intervals. **(A) **Weight of body, liver, spleen and heart. **(B) **Macrography of liver and spleen: seven days after the first poly (I:C) treatment. Mx-cre -: control mice (THOC5/FMIP (flox/flox)); Mx-cre +: THOC5/FMIP depleted mice (Mx-cre THOC5/FMIP (flox/flox)). **(C, D) **Seven days after the first poly (I:C) injection (3×), mouse spleens, heart, liver were fixed in formalin. Paraffin sections were stained by hematoxylin and eosin (C). Spleen and liver sections were supplied for immunohitochemical staining using cleaved caspase 3 (D). Original magnification: ×100 for C and ×200 for D.

To determine the pathology underlying gene knockout-induced death we analyzed specific organs and determined the weight of body, liver and spleen of five to six-week-old Mx-cre THOC5/FMIP (flox/flox) (n = 9) and THOC5/FMIP (flox/flox) (n = 6) mice before and after three times 250 μg poly (I:C) treatment. No significant differences were found in the weight of body or liver between poly (I:C)-treated and -untreated mice or between Mx-cre THOC5/FMIP (flox/flox) and control THOC5/FMIP(flox/flox) mice seven days after poly (I:C) injection (Figure 4A and 4B). Although there was no depression of THOC5/FMIP expression level, within seven days spleen weight of all Mx-cre THOC5/FMIP (flox/flox) mice dropped to 50% (*P *= 0.0002) of that in non-treated or control mice (Figure 4A). We next examined the histopathology of organs from the same mice (seven days after poly (I:C) injection), including, liver, heart, spleen and kidney.

Although white and red pulp in spleen are clearly present, periarterial lymphoid sheath (PALs) which includes the T-cell cuffs around the arteries and B-cell follicles appear to be reduced in favor of a parafollicular lymphocytic population which is interspersed more evenly in the red pulp. Throughout the organ, there are a few spots of proliferatively activated lymphoid cells (Figure 4C). Immunohistochemical staining using the cleaved caspase 3 specific antibody revealed that there were clearly more cleaved caspase 3 positive cells in clusters underneath the capsule (but not in the lymphoid follicles or PALs) in Mx-cre THOC5/FMIP (flox/flox) spleen than in control spleen (Figure 4C and 4D). In addition, no cleaved caspase 3 positive cells were observed in the red pulp, suggesting that some subpopulation of lymphocytic cells may be affected. Furthermore, liver, heart, and kidney, from THOC5/FMIP depleted mice were histologically normal throughout a 13 day period after poly (I:C) injection. Although the liver looked pale in these mice, no abnormalities were observed and no cleaved caspase 3 positive cells were seen in this organ (Figure 4D), suggesting that THOC5/FMIP depleted mice may have anemia, and/or internal bleeding.

Given the association of spleen with hematopoietic cells we then examined peripheral blood and bone marrow.

### THOC5/FMIP deletion causes severe leukocytopenia and anemia

**Figure 5:**
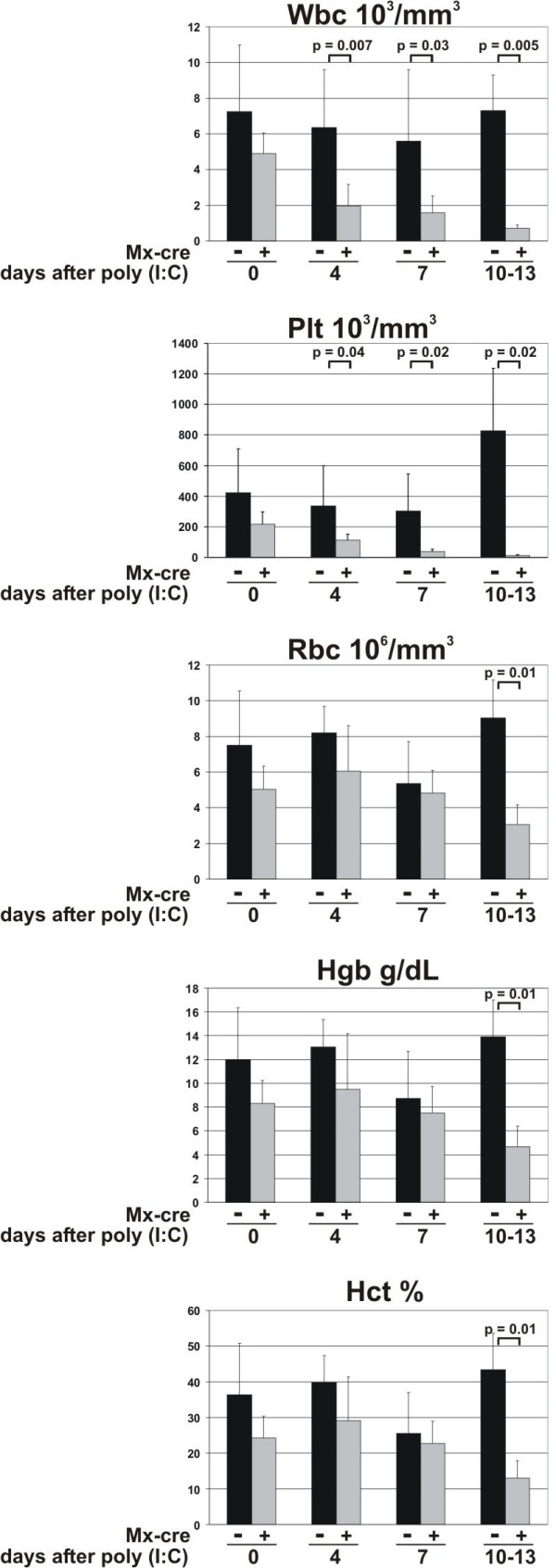
**The effect of deletion of THOC5/FMIP on hematopoiesis**. Six-week-old Mx-cre THOC5/FMIP (flox/flox) mice (Mx cre +, n = 9) and THOC5/FMIP (flox/flox) mice (Mx-cre -, n = 6) were injected with poly (I:C) (250 μg each) and blood was taken from the tail vein on zero, four and seven days after the first poly (I:C) injection. In addition, 10-13 days after poly (I:C) injection blood samples from Mx-cre THOC5/FMIP (flox/flox) mice (n = 3) which show severe symptom and control THOC5/FMIP (flox/flox) (n = 3) mice were taken from tails. Samples were analyzed on an ABC Vet automated blood counter. Wbc = white blood cells (10^3^/mm^3^); Plt = platelets (10^3^/mm^3^); Rbc = red blood cells (10^6^/mm^3^); Hgb = hemoglobin (g/dl); Hct = hematocrit (%).

Blood was taken from the tail vein of five-to-six-week old Mx-cre THOC5/FMIP (flox/flox) (n = 9) and THOC5/FMIP (flox/flox) (n = 6) mice on 0 (no poly I:C), four (2 × 250 μg (poly I:C)), seven (3 × 250 μg poly(I:C)), and 10 to 13 days (3 × 250 μg poly(I:C)) and examined. In agreement with previous reports, poly (I:C) injection induces slight leukocytopenia and thrombocytopenia in control mice within four days [22-24], however 10 to 13 days after poly (I:C) treatment, the number of leukocytes and platelets in control mice returned to normal (Figure 5). In THOC5/FMIP depleted mice, the number of leukocytes and platelets continued to decrease. After seven days, peripheral blood from most of the mice contained platelet numbers less than 40 × 10^3^/mm^3^, 13% (*P *= 0.02) of that seen in control mice after injection of poly (I:C). Simultaneously, we observed subdermal bleeding in these mice and within 13 days after the first poly (I:C) injection, all mice showed a further reduction of leukocytes and platelets (Figure 5). In addition, the erythrocyte count, hemoglobin and hematocrit levels went down to 2 to 4 × 10^6^/mm^3 ^(*P *= 0.01), 3-7 g/dl (*P *= 0.01), 13% (*P *= 0.01), respectively, suggesting that THOC5/FMIP depleted mice died from hematological disorders such as anemia, and internal bleeding. These data also agree with the observation that liver and spleen in THOC5/FMIP deleted mice appear pale.

### Depression of THOC5/FMIP gene causes cell apoptosis in bone marrow, but not of hepatocytes

**Figure 6:**
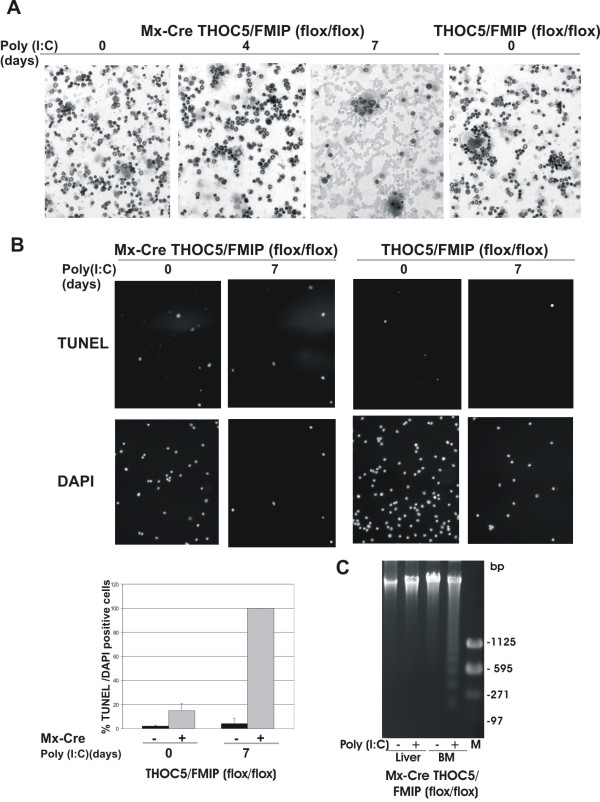
**Deletion of THOC5/FMIP causes apoptosis of leukocytes in bone marrow**. Six-week-old Mx-cre THOC5/FMIP (flox/flox) mice (n = 9) and THOC5/FMIP (flox/flox) control mice (n = 6) were injected with poly (I:C) (250 μg each) and the femora were then isolated. **(A)**: Bone marrow cells were spun down onto glass slides and then stained with May-Grunwald Giemsa and hematoxylin. **(B)**: Aliquots of same preparation were stained with TUNEL and DAPI. Results are the mean +/- SEM of %TUNEL positive/DAPI positive cells (n >2000 cells). Original magnification: ×200 for all panels. **(C)**: Aliquots of 2-3 μg of DNA from liver and bone marrow of poly (I:C) treated (+) and untreated (-) Mx-cre THOC5/FMIP (flox/flox) mice were separated on 1.5% (w/v) agarose gel, stained with ethidium bromide (2 μg/ml) and photographed under UV light. M: base pair Marker.

As severe leukocytopenia and anemia were observed in THOC5/FMIP depleted mice, we examined the bone marrow cells after induced depletion of THOC5/FMIP protein. Bone marrow cells from five to six-week-old mice were flushed from femora zero, four or seven days after the first 250 μg poly (I:C) injection (n = 9). Cytospin preparations were stained by May Grunwald solution and hematoxylin. Although after four days we did not detect any difference in morphology of bone marrow cells, after seven days it became apparent that few hematoxylin-stained cells were present in samples from Mx-cre THOC5/FMIP(flox/flox) mice (Figure 6A). A high proportion of those cells that were present showed dense chromatin staining, reminiscent of apoptotic cells. To examine whether the cells that survived were apoptotic more than 2,000 4', 6-Diamidino-2-phenyindole (DAPI) positive bone marrow cells were co-stained with terminal deoxynucleotidyl transferase-mediated dUTP nick end labeling (TUNEL) and DAPI for each preparation. Two and four percent respectively of DAPI positive bone marrow cells in control THOC5/FMIP(flox/flox) mice are stained with TUNEL in the presence or in the absence of poly (I:C) treatment (Figure 6B). In Mx-cre THOC5/FMIP(flox/flox) mice, 15% of bone marrow cells are stained without poly(I:C) injection, possibly as a result of the natural presence of interferons. After three poly (I:C) injections, only a few DAPI positive cells are left and all remaining cells were stained with TUNEL (Figure 6B), suggesting that deletion of THOC5/FMIP causes bone marrow cell apoptosis. Furthermore, no significant increase of TUNEL positive cells (10-20%) was observed, suggesting that apoptotic cells may fail to stay in bone marrow or be phagocytosed. DNA laddering, indicative of apoptosis, in bone marrow cells and hepatocytes was also examined. We detected DNA fragmentation associated with apoptosis from the poly (I:C) injected bone marrow cells, but not from the liver of the same mouse (Figure 6C).

Many erythrocytes are still present in bone marrow after seven days. Given their extended lifetime compared to white cells it is probable that the steady decrease in their numbers is due to decreased rates of blood cell production in the marrow.

The half life of erythrocytes in mice is about 18 days [25,26]. The extent of the effect we observe (of about a 40% reduction over 10-13 days) argues for dysfunctional erythropoiesis as the causal effect. However both white cell and red cell numbers are depleted. Therefore the lesion leading to death may be associated with hematopoiesis.

### Hematopoietic progenitor cell populations are depleted upon THOC5/FMIP deletion

The marrow hypoplasia observed in Mx-cre THOC5/FMIP (flox/flox) mice after poly (I:C) injection led us to consider the effects on hematopoietic progenitor cells and a population enriched for primitive hematopoietic cells including long-term reconstituting cells. To examine hematopoietic progenitor cells, bone marrow cells were isolated from mice four days after poly (I:C) injection. Since we could obtained more consistent data from mice treated with 2 × 500 μg than mice treated with 2 × 250 μg at this time point, we injected five to six-week-old Mx-cre THOC5/FMIP (flox/flox) (n = 5) and THOC5/FMIP (flox/flox) (n = 6) mice with (2 × 500 μg (poly I:C) at a two-day interval) in this experiment.

**Figure 7:**
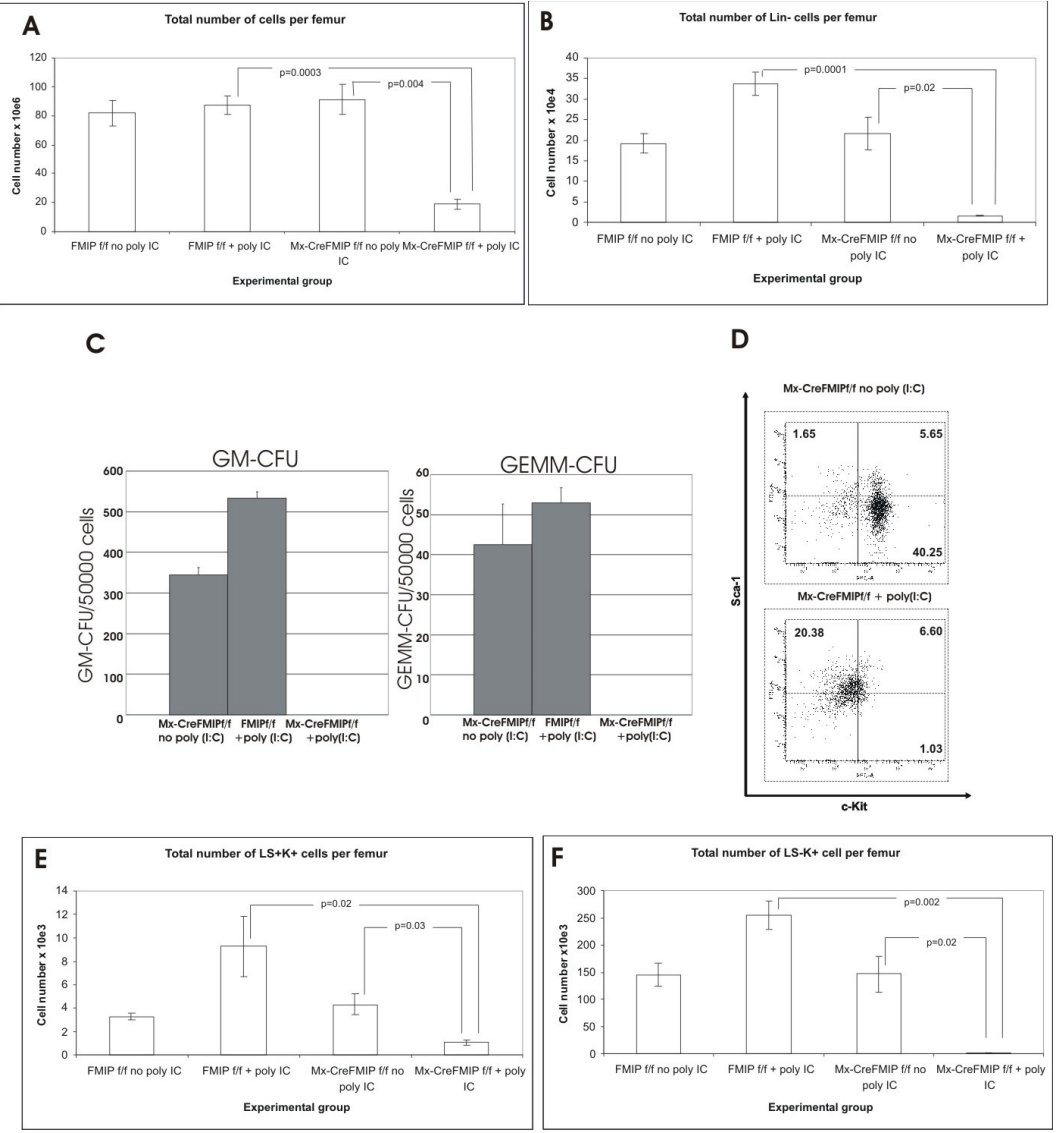
**Deletion of THOC5/FMIP causes loss of primitive hematopoietic cells from the bone marrow**. Five-to-six-week-old Mx-cre THOC5/FMIP (flox/flox) (n = 5) and THOC5/FMIP (flox/flox) (n = 6) mice were injected with poly (I:C) (2 × 500 μg, two days interval). Then, bone marrow cells were isolated from mice four days after poly (I:C) injection. The effect of THOC5/FMIP depletion on the total numbers of primitive cells present in the bone marrow was assessed using a number of different approaches (A; B): total bone marrow cellularity per femur was assessed (A) and the number of cells present in the bone marrow which do not express lineage markers (B) (and therefore have a primitive phenotype) was calculated (Mean+/-SEM). Results shown are for Mx-cre THOC5/FMIP (flox/flox) (Mx-CreFMIPf/f) mice with (n = 5) and without (n = 4) poly (I:C) (2 × 500 μg, two day interval), and THOC5/FMIP (flox/flox) (FMIPf/f) control mice with (n = 6) and without (n = 6) Poly (I:C) (2 × 500 μg, two day interval) (C). The number of colony forming cells per femur was determined for Colony Forming Unit-Granulocyte macrophage (GM-CFU) and Colony Forming Unit-Granulocyte Erythroid Macrophage Megakaryocyte (GEMM-CFU). Results shown are the mean+/-SEM, n = 3. (D): Flow cytometric profile of Kit and Sca staining in Lineage marker depleted cells from mice treated with and without poly (I:C) (3 × 250 μg, two day interval). The results shown are representative of six experiments. (E; F) Results shown are for Mx-cre THOC5/FMIP (flox/flox) mice with (n = 5) and without (n = 4) poly (I:C) (2 × 500 μg, two day interval), and THOC5/FMIP (flox/flox) control mice with (n = 6) and without (n = 6) poly (I:C) (2 × 500 μg, two-day-interval), and are expressed as total number of LSK cells (E) and Lin-Sca-Kit+ cells (LS-K+) cells (F) per femur, error bars show SEM.

First we determined femoral cellularity which was found to be depressed in Mx-cre THOC5/FMIP (flox/flox) mice treated with poly (I:C) (Figure 7A). The same samples were then taken for assessment of primitive hematopoietic cell numbers. We determined the total number of cells which were not expressing lineage markers for mature hematopoietic cells, a measure of progenitor cells present in the marrow. The Lin- cells were depleted to a greater extent in THOC5/FMIP depleted animals than in control animals (Figure 7B). We therefore assessed multipotent colony forming cell numbers in soft gel assays. We could detect no GEMM-CFU (granulocyte, erythrocyte, monocyte, macrophage-colony forming unit) (which includes early erythroid progenitors, see below) in THOC5/FMIP depleted murine femora whilst they were present in both control populations assayed. Similarly, granulocyte macrophage colony forming cells (GM-CFU) were undetectable in THOC5/FMIP depleted bone marrow but were present in control femora (Figure 7C). The effect of poly (I:C) injection in Mx-cre THOC5/FMIP(flox/flox) mice on primitive multipotent colony forming cells, GEMM-CFU, and GM-CFU is therefore a profound reduction. Furthermore, an analysis of the Lineage marker depleted, Sca^+^, Kit^+ ^(LSK) cell numbers present in the bone marrow revealed that the proportion of LSK cells (which co-enrich with primitive hematopoietic cells and include long term reconstituting cells) was not substantially changed when Mx-cre THOC5/FMIP(flox/flox) mice were treated with poly (I:C). However the total number of these cells per femur was decreased by 80% per femur. This argues for primitive hematopoietic cells as well as lineage specific progenitor cells being depleted as a consequence of THOC5/FMIP knockout.

As detailed the Lineage marker negative cell population, generally considered a progenitor cell cohort, was reduced by 92% by poly (I:C) treatment of Mx-cre THOC5/FMIP(flox/flox) mice (Figure 7B) and myeloid progenitors are decreased. Furthermore, lineage marker depleted Sca- Kit+ cell population which consists largely of erythroid progenitors [27] was also decreased by 98% (Figure 7F). We envisage the stem cell compartment is apparently radically but slightly less affected (74% decrease, Figure 7E) as it turns over more slowly than rapidly cycling progenitor cell populations. These data are consistent with a failure of hematopoiesis in the stem cell and progenitor cell compartments as a result of FMIP levels falling in hematopoietic cells.

### A transfer of normal bone marrow cells rescued 70% of mice from death by THOC5/FMIP depletion

**Figure 8:**
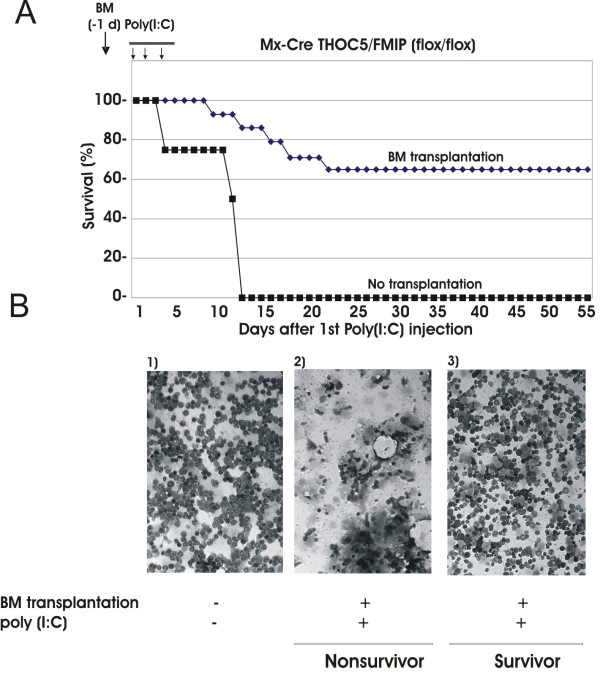
**Bone marrow transplantation rescues THOC5/FMIP depleted mice from death**. **(A) **Bone marrow (BM) transplantation: 10^6 ^bone marrow cells obtained from THOC5/FMIP (flox/flox) mice (n = 14) were transferred into six-to-eight-week-old nonablated Mx-cre THOC5/FMIP (flox/flox) mouse for each and one day after transplantation recipient mice were injected with 250 μg of poly (I:C). Injection was performed i.p. three times at two-to-three-day intervals. No transplantation: Mx-cre THOC5/FMIP (flox/flox) mice without BM transplantation were injected with poly (I:C). **(B) **Bone marrow cells were spun down onto glass slides and then stained with May-Grunwald Giemsa and hematoxylin. Bone marrow cells were derived from 1) non-treated Mx-cre THOC5/FMIP (flox/flox) mouse. 2) a poly (I:C) injected normal bone marrow cell recipient Mx-cre THOC5/FMIP (flox/flox) mouse which died 18 days after the first injection (Nonsurvivor). 3) a poly (I:C) injected normal bone marrow cell recipient Mx-cre THOC5/FMIP (flox/flox) survivor (eight weeks after the first injection) (Survivor).

Although THOC5/FMIP was depleted in liver, kidney, heart and bone marrow, the only evident phenotype alteration was observed in bone marrow cells. It may be explained that THOC5/FMIP depletion displays a phenotype first in this fast turnover tissue that is very dependent on stem cell self-renewal and differentiation. Hematopoietic cells especially white cells such as neutrophils turn over rapidly, and it is in keeping with observations on the sensitivity of hematopoiesis insult such as irradiation or cytotoxic drugs that the first major signs of THOC5/FMIP removal are observed in bone marrow derived cells. The question rose as to whether other organs, such as liver, kidney and heart would have shown pathological alterations if the mice had survived for longer periods. Therefore, we examined next whether a transfer of normal bone marrow cells rescues THOC5/FMIP depleted mice. We transferred 10^6 ^bone marrow cells derived from THOC5/FMIP(flox/flox) mice into nonablated Mx-cre THOC5/FMIP(flox/flox) mice via i.v. and a day after transplantation we injected recipient mice with poly (I:C), followed by two additional injections. Nine out of 14 mice survived longer than two months and no symptoms were observed (Figure 8A). Furthermore, the exon IV/V of THOC5/FMIP gene was deleted completely from liver genomic DNA of survivors (data not shown), indicating that cre-recombinase was expressed by poly (I:C) injection in liver. Although THOC5/FMIP expression was drastically down-regulated in the livers of survivors, histological abnormalities were not observed in liver, kidney and heart. Cytospin preparations revealed that only few hematoxylin-stained cells were present in samples from four bone-marrow-recipient mice which died (Figure 8B, Non-survivor), while normal populations of hematoxylin-stained cells with few apoptotic cells were present in samples from survivors (Figure 8B, Survivor). The phenotype of the non-survivors is identical (leukocytopenia, anemia, bleeding, the reduced size spleen, and so on.) to that of non-recipient mice, suggesting that bone marrow transplantation did not work in these mice. It may be related to the fact that the bone marrow cell transplantation was performed into nonablated mice. In some case, there may be no *open space *for donor cells [28]. Another possibility is that each Mx-Cre FMIP flox/flox mouse has a slightly different strain background, suggesting that in some cases, a graft-versus-host reaction may occur.

All bone marrow cell recipient mice showed extramedullary hematopoiesis in spleen, but no significant pathological change was observed in other organs. These data suggest that THOC5/FMIP is required in bone marrow for hematopoiesis, however THOC5/FMIP is less important in other organs, such as liver and heart.

### Deletion of THOC5/FMIP results in markedly decreased THOC1

**Figure 9:**
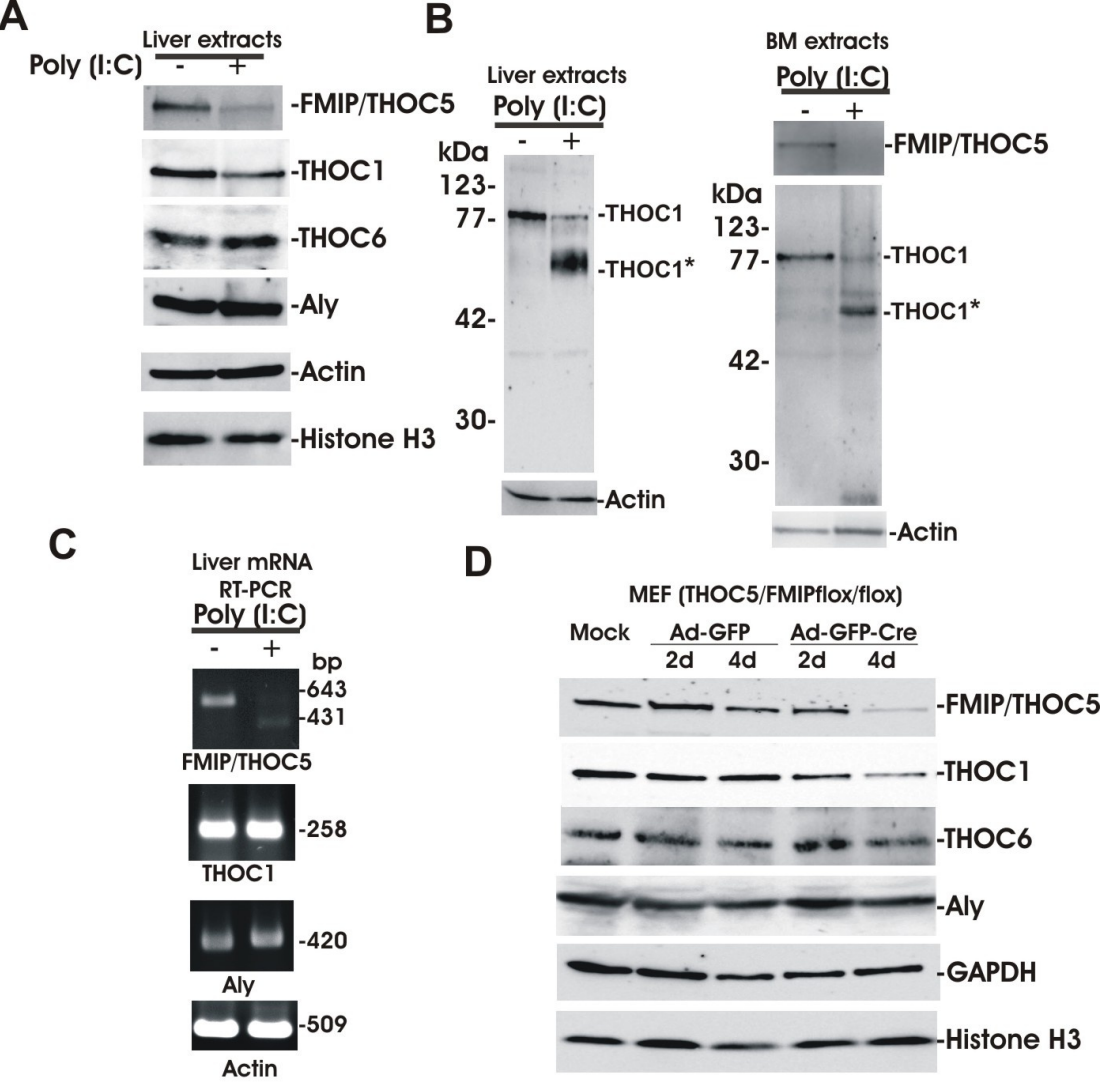
**Depletion of THOC5/FMIP causes down-regulation of THOC1**. (A): Liver extracts from three times poly (I:C) 250 μg treated (+) (14 days) and untreated (-) six-week-old Mx-cre THOC5/FMIP (flox/flox) mice (n = 3) were examined by THOC1, Aly, THOC6, THOC5/FMIP, Actin and Histone H3 specific immunoblotting. Each sample consisted of 100 μg of liver. (B): Liver or bone marrow extracts from poly (I:C) treated (+) (14 days) were examined by THOC1, THOC5/FMIP, and actin specific immunoblotting. THOC1*: 60 kDa THOC1 degradation product from poly (I:C) treated liver or bone marrow sample. (C): mRNA was isolated from the same liver samples and THOC5/FMIP (643 bp and 431 bp (without exons IV/V)), THOC1 (258 bp), Aly (420 bp) and Actin (509 bp) specific RT-PCR were performed. (D): MEF THOC5/FMIP (flox/flox) cells were infected with adenovirus carrying GFP gene (Ad-GFP) or adenovirus carrying GFP and cre-recombinase genes (Ad-GFP-Cre) (M.O.I. 50; Vector Biolab, Philadelphia, PA, USA) then lysed by laemmli buffer. Extracts were then examined by THOC1, Aly, THOC6, THOC5/FMIP, GAPDH and Histone H3 specific immunoblotting.

We have recently shown that the C-terminal domain of THOC5/FMIP forms a complex with THOC1 and the N-terminal domain binds to THOC7 directly [19]. Furthermore, the binding of THOC7 to THOC5/FMIP requires the nuclear localization of THOC7. These facts raise the question as to whether loss of THOC5/FMIP molecule influences stability of another member of the complex. Since THOC5/FMIP expression was drastically down-regulated in the livers, we analyzed the expression of the members of the THO complex in liver tissue before and after poly (I:C) injection. Fourteen days after poly (I:C) injection, there was more than an 80% decrease of THOC5/FMIP observed at the protein level. Although Aly, and THOC6 were unchanged, the amount of THOC1 went down to 30% within 14 days (Figure 9A). A reduced level of THOC1 was also observed in THOC5/FMIP depleted bone marrow cells (Figure 9B). Furthermore, a broad band with molecular mass of 60 kDa was detected from poly (I:C) treated liver and bone marrow cell extract by THOC1 specific immunoblot analysis (Figure 9B), suggesting that THOC1 protein may not be stable in the absence of THOC5/FMIP. To examine whether THOC1 decreases at the mRNA level, we performed RT-PCR in these organs. Using THOC5/FMIP specific primers 643 bp product was obtained from untreated liver mRNA, while a 431 bp product was obtained from poly (I:C) treated liver mRNA (Figure 9C). The difference in size corresponds to the exon IV/V deletion in treated animals. The mRNA levels of THOC1 and Aly were unchanged by treatment with poly (I:C). To confirm these data without poly (I:C) effect, we utilized mouse embryo fibroblasts (MEF) isolated from THOC5/FMIP (flox/flox) mice. After infection with adenovirus carrying GFP and cre-recombinase, THOC5/FMIP was drastically reduced within four days. In agreement with data obtained from liver tissue, the level of THOC1 protein was also drastically reduced (Figure 9D). As control we infected adenovirus carrying GFP alone. We did not observe a reduction of THOC5/FMIP or THOC1 in control virus infected MEF. These data suggest that depletion of THOC5/FMIP causes down regulation of THOC1 also in cell culture system.

Our data indicate that THO complex plays a key role in early embryogenesis and the THO complex in bone marrow is essential for hematopoiesis, however it is less important in differentiated cell types such as hepatocytes and heart muscles.

## Discussion

We have previously shown that depletion of THOC5/FMIP by siRNA or ectopic expression causes abnormal hematopoiesis and abnormal adipocyte differentiation in myloid progenitor or mesenchymal progenitor cell lines [14-17]. Furthermore, although THO complex does not play a role in nuclear export of bulk mRNA, THOC1 deletion causes embryonic lethality [13]. This led us to investigate the potential roles for THOC5/FMIP *in vivo*. To do this we first generated conventional knockout mice, deleting THOC5/FMIP. These mice died at an early embryonic stage. Furthermore, although heterozygotic THOC5/FMIP deletion mutant mice were fertile and did not display any phenotypic or histological abnormalities after birth, 50% of THOC5/FMIP heterozygotes died during embryogenesis (El Bounkari and Tamura, unpublished data), suggesting that the level of expression of THOC5/FMIP plays a key role in embryo development.

When an inducible system was developed a key developmental system, hematopoiesis, was found to be sensitive to depletion of THOC5/FMIP. Our data showed that bone marrow cells and spleen cells, but no other organs, such as liver, heart, kidney, intestine, testicle, or lung, became apoptotic in THOC5/FMIP deficient adult mice, suggesting hematopoietic required THOC5/FMIP. In addition, transplantation with bone marrow cells derived from cre minus THOC5/FMIP(flox/flox) mice rescues poly (I:C) injected Mx-cre THOC5/FMIP(flox/flox) mice from death. This extends back to the primitive cells in the hematopoietic system, since THOC5/FMIP depleted mice showed a major decrease in progenitor cells (GEMM-CFU, GM-CFU, Figure 7C) and also a fall in the slowly cycling bone marrow LSK cells (Figure 7D). The effects of an RNA processing protein on stem cells were also profound and this we argue in part explains the massive effect on the committed progenitor cell compartment compared to LSK cells. Nonetheless, the rapid onset of the effect also argues that the progenitor cell compartment is sensitive to loss of FMIP/THOC5 expression.

Mx-cre directed depletion by poly I:C injection was observed in bone marrow cells, heart, kidney, liver and spleen [21,29]. Indeed, THOC5/FMIP specific PCR analyses using genomic DNA as a template revealed that exons IV/V were deleted from these organs, including spleen after only one × times poly (I:C) injection. We did not observe the depletion of THOC5/FMIP protein in spleen for any time period however, the size of spleen is drastically reduced after seven days poly (I:C) injection, suggesting that apoptotic cells are released from spleen and that the THOC5/FMIP protein half life in certain cells was longer than in other cells.

The issue remains how and why THOC5/FMIP is required for primitive hematopoietic as well as embryo stem cell survival and/or proliferation. Our data suggest that the THO complex is required for mRNA export of particular genes that play a key role in hematopoietic primitive (multipotent) and committed progenitor cell survival and/or proliferation. Alternatively, the THO complex may be essential for bulk poly (A)+ RNA export in primitive cells. The yeast TREX complex is composed of the THO transcription elongation complex (Hpr1 (the THOC1 orthologue), Tho2 (the THOC2 orthologue), Mht1 and Thp2), Tex1 (the THOC3 orthologue), Sub2 (the UAP56 orthologue) and Yra1 (the Aly/THOC4 orthologue) [30]. Yeast TREX mutants show a nuclear export defect for bulk poly A+ RNAs and are synthetically lethal with many mutants of the mRNA machinery [30]. In Drosophila loss of the THO complex function results in only minor differences in transcription profiles as revealed by the whole genome array [5]. The analysis of the cytoplasmic mRNA from bone marrow cells was not successful, because cytoplasmic mRNA from bone marrow cells was not stable enough for microarray analysis. Therefore, we analyzed the expression level of cytoplasmic mRNA in fibroblasts. In the presence or absence of THOC5/FMIP, we found that less than 100 genes were down-regulated more than three-fold using the mouse whole genome array (Hauser and Tamura, unpublished data). In the fibroblast system, however, the depletion of THOC5/FMIP did not cause apoptosis, but reduced cell growth (Tran and Tamura, unpublished data), suggesting that THOC5/FMIP has a specific function in proliferating cells. It has been shown recently that in response to treatment of mice with interferon-alpha hematopoietic stem cells efficiently exit G_0 _and enter an active cell cycle [31], suggesting that poly (I:C) treatment may exacerbate the phenotype which is observed in THOC5/FMIP deletion mice. Further studies will be required to examine whether the mice would present the same phenotype if other methods of conditional deletion of THOC5/FMIP in the relevant cell types were used.

We show here that the THOC5/FMIP binding partner, THOC1 was down-regulated via THOC5/FMIP knockdown. Furthermore, we have shown that the expression of mutant THOC5/FMIP lacking the THOC1 binding site degraded faster than the wild-type and down-regulated the level of endogenous THOC1 [19], suggesting that these two molecules may stabilize each other forming a functional unit. It indicates that the phenotype we observed might be the result of both THOC1 and THOC5/FMIP depletion. It has been shown that over-expression of THOC1 causes apoptosis in several cells [12]. The deletion of THOC1 also causes apoptosis in cancer cells, but not in normal fibroblasts [12]. Furthermore, the embryonic development of conventional THOC1 knockout mice is arrested around the time of implantation [13]. Interestingly, THOC1 possesses the death domain and interacts with Rb protein, suggesting that THOC1 may involve the apoptosis signal directly [32].

Our analysis demonstrates a link between THOC5/FMIP protein function and the normal developmental processes seen in adult hematopoiesis. We now have a suitable model system to determine how THO complex proteins act, enabling primitive cell survival and proliferation.

## Conclusions

THOC5/Fms interacting protein is an essential element in the maintenance of hematopoiesis. Furthermore, mechanistically depletion of THOC5/Fms interacting protein causes the down-regulation of its direct interacting partner, THOC1 which may contribute to altered THO complex function and cell death.

## Methods

### Generation of THOC5/FMIP deficient mice

THOC5/FMIP deficient mice were generated using a genomic DNA fragment containing exons II, III and IV, isolated from a 129/ola genetic background. The targeting vector was constructed from a 3.9 kb PCR fragment and an adjacent 2.2 kb fragment harboring exons IV/V. Fragments were introduced into the pPNTloxPneo vector via NotI, XhoI restriction sites and KpnI, respectively. An additional loxP site was inserted into the SacI site downstream of exon V (Figure 1A). ES cells were electroporated with a NotI linearized target vector and grown under double selection as previously described [33]. ES cell clones carrying the inserted floxed neo cassette and floxed THOC5/FMIP exons IV/V were identified by the presence of an 1.9 kb BamHI restriction fragment in Southern Blot analysis with an external 3' probe (Figure 1B). One clone was used for injection into C57Bl/6 blastocysts to generate germ line chimeras. Chimeras were mated with wild type mice to obtain heterozygous floxed offspring.

### Genotyping for THOC5/FMIP (flox/flox) and Mx-cre THOC5/FMIP (flox/flox) mic

Genotyping was performed from tail-derived sample DNA extracted with Viagen DirectPCR-Tail (Viagen, Erlangen, Germany) by PCR. THOC5/FMIP primers flanked the floxed region with 5'-CCCTCGGCCCCTTTTGAG-3' and 5'-CAGCACTGGAGCGGGAGATGT-3'. Corresponding bands gave 177 bp (wt allele) and 210 bp sizes (floxed allele), respectively. Homozygous offspring were interbred to generate THOC5/FMIP(flox/flox) mice and the resulting genotypes were determined by PCR. After crossing with Mx-cre deleter mice, mice were genotyped by PCR using primers Cre1 5'-CCGGGCTGCCACGACCAA-3' and Cre2 5'-GGCGCGGCAACACCATTTT-3' for cre gene and primers for floxed THOC5/FMIP gene. After poly (I:C) injection, mice were genotyped by PCR using primers 5'-TGCTGGCATTGAACTGTG-3' and 5'-CAGCACTGGAGCGGGAGATGT-3' for the determination of exons IV/V deletion.

### RT-PCR analysis

RNA was isolated from mouse liver tissue with the Qiagen RNeasy kit (Qiagen, Hilden, Germany) according to the manufacturer's recommendations. Reverse transcription was carried using oligo dT primers and Omniscript reverse transcriptase kit (Qiagen) following the instructions provided. For PCR the following primer pairs were used:

THOC1: forward primer: 5'-CTCACTTCTTCAGCCAA CC-3'; reverse primer: 5'-AAGGAGCCAAAATCTTCCAT-3' (product size = 258 bp), Aly: forward primer: 5'-CTGGACTTCGGAGTGTCAGATGC-3'; reverse primer: 5'-CCTTGCATTGTA AGCATCCAGC-3' (product size = 420 bp), THOC5/FMIP: forward primer: 5'-TCT GCCTTTTCACCTGGAAG-3'; reverse primer: 5'-CTCGGTACTTTTCTGCCA GC-3' (product size = 643 bp (wild type), or 431 bp (without exons IV/V)), beta-actin forward primer: 5'-AACACCCCAGCCATGTACGTAG-3'; reverse primer: 5'-GTGTTGGCATAGAGGTCTTTACGG-3' (product size = 509 bp). PCRs were set up according to the following profile: an initial denaturation step of 94°C for three minutes, 35 cycles of 94°C for 30 seconds, 60°C for 30 seconds, and 72°C for 30 seconds followed by a final extension step at 72°C for 10 minutes. Separation of the DNA fragments was carried out on 1.5% (w/v) agarose gels, stained with ethidium bromide (2 μg/ml) and photographed under UV light.

### Poly (I:C) injection

Fifty, 250 or 500 μg of poly(deoxyinosinic/deoxycytidylic) acid (Invivogen, San Diego, CA, USA) were injected intraperitoneally into three day-, six week- and nine week-old mice respectively, three times at two-to-three-day intervals.

### Hematopoiesis assays

Cytospin preparations were generated from bone marrow cells then visualized by May-Grunwald and Hematoxylin stain. Peripheral blood was collected by means of tail venipuncture into microcapillary tubes pre-coated with ethylenediamine tetraacetic acid. Samples were analyzed on an ABC Vet automated blood counter (ABX Hematology Inc. Garden Grove, CA, USA).

Hematopoietic lineage marker depleted (Lin-) cells were obtained using a cocktail of lineage marker antibodies (Pharmingen, Oxford, UK). Sheep anti-rat antibody magnetic beads (Dynal, Wirral, UK) were employed to deplete cells expressing lineage markers. Lineage depleted cells were then labeled with fluorescein isothiocyanate-labelled Sca-1 antibody and with phycoerythrin labeled c-Kit antibody (Becton Dickinson, Oxford, UK). The expression of Sca antigen and Kit antigen were employed to define primitive cells present in the bone marrow using flow cytometry with a Becton Dickinson FacsAria instrument (Becton Dickinson, Oxford, UK) [34].

Colony forming assays were performed using soft gel assays from Stem Cell Systems (Vancouver, BC. Canada) following the manufacturer's instructions.

### TUNEL, DAPI staining and DNA ladder

To assess the degree of apoptosis, an *in situ *cell death detection kit (Roche, Mannheim, Germany) was used for terminal deoxynucleotidyl transferase-mediated dUTP nick end labeling (TUNEL) staining and 4',6-diamidino-2-phenylindole (DAPI) (Sigma, Munich, Germany) was used for control DNA staining. Genomic DNA was isolated from liver and bone marrow cells from poly (I:C) treated and untreated mice, using the DNeasy Tissue Kit (Qiagen). Aliquots of 2 to 3 μg of DNA were separated on 1.5% (w/v) agarose, stained with ethidium bromide (2 μg/ml) and photographed under UV light.

### Immunohistochemistry

Immunohistochemistry study was performed as detailed previously [35].

### Isolation of mouse embryo

Mouse embryos were isolated under the microscope according to a protocol from Beckers et al. (2007) [36].

### Western Blot procedures

Mouse organs were extracted with lysis buffer containing 10 mM Tris HCl pH 7.6, 50 mM NaF, 1 mM PMSF, 10 mM EDTA, 1%(w/v) Triton-X 100, 8 M Urea, 1%(w/v) Trasylol (Bayer Vital, Leverkusen, Germany). Details of immunoblotting have been described previously [37]. Polyclonal antibody against actin, monoclonal antibodies against Aly/THOC4, THOC6 and GAPDH were purchased from Santa Cruz biotechnology inc.(Santa Cruz, CA, USA), rabbit antibodies against Histone H3 and cleaved caspase 3 were from Cell Signaling Technology (Beverly, MA, USA), monoclonal antibody against THOC1 (p84 N5) was from Gene Tex. Inc (San Antonio, TX, USA), monoclonal antibody against THOC5/FMIP was generated as described previously [16]. Corresponding proteins were visualized by incubation with peroxidase conjugated anti-goat, mouse or rabbit immunoglobulin followed by incubation with SuperSignal West FemtoMaximum Sensitivity Substrate (Pierce, Rockford, IL, USA). Results were documented on a LAS3000 (Fujifilm, Kanagawa, Japan). Signal intensity of chemoluminescence was quantified using TINA 2.0 software (Raytest Isotopenmessgeraete GmbH, Straubenhardt, Germany).

## Abbreviations

DAPI: 4', 6-Diamidino-2-phenyindole; ES: embryonic stem; GEMM-CFU: granulocyte, erythrocyte, monocyte, macrophage-colony forming unit; GM-CFU: granulocyte-macrophage colony forming unit; TAP: tip associating protein;THO: suppressors of the transcriptional defects of *hpr 1delta *by overexpression; THOC: THO complex; TREX: transcription/export; TUNEL: terminal deoxynucleotidyl transferase-mediated dUTP nick end labeling.

## Authors' contributions

AM and SCNS carried out the poly (I:C) injection, RT-PCR, Immunoblot, cytospin preparation, DNA ladder, and prepared figures, RP generated THOC5(flox/flox) mice, OEB carried our the blood analyses, SKF supervised the maintenance of mouse colony, and performed genotyping. AK participated in the design of the study, supervised the study and analyses of data. ADW participated in its design and coordination, and review the manuscript. EJ and ES carried out FACS analysis and hematopoietic colony assay. ADG performed the pathological analyses. TT participated in the design of the study, contributed to the data analysis, and wrote and finalized manuscript. All authors participated in the discussion and approved the final manuscript.
